# Rising scabies incidence and the growing burden on GPs: a retrospective longitudinal study

**DOI:** 10.3399/BJGPO.2025.0037

**Published:** 2025-12-19

**Authors:** Saskia C van der Boor, Ines Figaroa, Annemarie A Uijen, Stijn FH Raven, Cornelia HM van Jaarsveld

**Affiliations:** 1 Radboud University Medical Center, Research Institute for Medical Innovation, Department of Primary and Community Care, Nijmegen, Netherlands; 2 Department of Infectious Diseases, Public Health Service region Utrecht, Zeist, Netherlands

**Keywords:** epidemiology, scabies, general practice

## Abstract

**Background:**

Scabies cases are rising in several high-income European countries, but its true incidence remains unclear owing to its non-notifiable status. GPs play a key role in managing scabies, yet the impact on primary care is unknown.

**Aim:**

To assess the epidemiology and care burden of scabies-related episodes in general practice.

**Design & setting:**

A retrospective longitudinal study using pseudonymised data from Dutch GPs.

**Method:**

Data from five general practices, covering nearly 40 000 patients from 2014–2023, were analysed. Scabies-related episode incidence, time between scabies-related symptoms and first GP encounter (patient’s delay), time between first GP encounter and scabies diagnosis (doctor’s delay), and care burden were compared between two periods: 2014–2020 (low incidence) and 2021–2023 (high incidence).

**Results:**

In total, 1525 scabies-related episodes, including 4555 recorded encounters, were included. Between 2014 and 2023, the incidence increased from 99 to 1341 episodes per 100 000 patient-years, with encounters rising from 614 to 2438 per 100 000 patient-years between the two periods. The largest increase in encounters occurred in those aged 17–25 years (*P*<0.001) and in females (*P* = 0.002). Patient delays >28 days decreased from 26.4% to 14.3%. Scabies diagnoses during initial consultations improved from 84.9% to 92.3%. Encounters without interventions declined from 36.9% to 14.4%. Medical prescriptions and referrals per 100 000 patient-years increased.

**Conclusion:**

Our findings highlight an exponential increase in scabies-related episodes, significantly increasing the burden on general practices. Regions experiencing rising scabies incidence rates should prioritise public health interventions to curb transmission, which require a coordinated clinical and public health response.

## How this fits in

The rising incidence of scabies in European countries remains largely unquantified owing to its non-notifiable status. In this study, we demonstrate for the first time that this trend significantly increases the burden of care in general practices. Clinicians and public health experts should prioritise public health interventions to address this growing scabies-related burden.

## Introduction

Scabies is a skin infestation caused by the mite *Sarcoptes scabiei* variant *hominis*, which lays eggs in the upper layer of the skin and causes a pruritic skin eruption.^1^ Complications may occur when the skin’s protective barrier function is interrupted, promoting secondary bacterial infections.^1,2^ Transmission primarily occurs through prolonged skin-to-skin contact but may also occur indirectly through contaminated items such as bedding and clothing.^3,4^ In 2017, the World Health Organization listed scabies as a neglected tropical disease and estimated that at least 200 million people worldwide suffer from scabies at any given time, with prevalence estimates in countries ranging from 0.2% to 20%–30% in the general population in regions in the Pacific.^5^ Although the burden of scabies is highest in low-income countries,^2^ an increasing number of scabies cases over the past 10–20 years has been reported in multiple European countries as well, including Germany, France, Norway, Croatia, Spain, and the Netherlands.^6–10^ However, the true prevalence is unknown as scabies is not notifiable in most European countries.^11^ In the Netherlands, only cases of crusted scabies and scabies cases in vulnerable populations in institutional settings, such as daycare centres and healthcare institutions, are required to be reported to the Dutch Public Health Service (PHS). This limited reporting makes it difficult to estimate the true incidence of scabies and its impact on healthcare demand.

In individual patients presenting with scabies-related complaints, GPs play a central role in the diagnosis and management of episodes. As the first point of contact within the healthcare system, GPs are responsible for the initial assessment of patients, which may include a medical history, physical examination, and diagnostic procedures to establish a diagnosis. Based on the clinical findings, the GP initiates appropriate treatment at the index consultation by prescribing scabies medication and provides advice for close contacts. In cases where the condition is complex or unresponsive to standard treatment or the diagnosis is unclear, a patient may be referred to a dermatologist for further evaluation and management.

Given the central role of GPs in managing scabies-related complaints, the primary objective of this study is to describe the epidemiology and burden of care of scabies-related episodes in selected Dutch general practices between 2014 and 2023. We examine healthcare utilisation among patients with scabies-related symptoms and the evolving patterns of scabies management by GPs over time, aiming to better estimate the impact of rising scabies incidence on healthcare services.

## Method

### Study population

In this retrospective longitudinal study, we utilised pseudonymised data from the Dutch Family Medicine Network (FaMe-Net), the world’s oldest functioning Practice-Based Research Network (PBRN) from the Radboud University Medical Center in Nijmegen, the Netherlands. FaMe-Net systematically records health problems and corresponding primary care interventions within defined episodes of care using International Classification of Primary Care (ICPC-2). In 2022, FaMe-Net contained data of more than 42 000 listed patients, derived from six family practices and 35 GPs.^12,13^ Here, we included data from five practices, which had data from 1 January 2014 onwards. From these practices, containing detailed data from almost 40 000 patients, all episodes with the ICPC-2 code ‘S72: infestation mites’ between 1 January 2014 and 31 December 2023 were included. Additionally, we also included episodes without a formal scabies diagnosis but with prescribed scabies medication (permethrin or ivermectin). If a patient had multiple episodes, data from all episodes were incorporated, including those with the subcode S72 (suspected scabies, but ultimately diagnosed with another condition). Registered follow-up contacts from episodes starting between 2014 and 2023 were included up to March 2024.

### Data

For the purpose of this study, we extracted pseudonymised structured data from scabies-related episodes of care from electronic patient records, including data related to patient characteristics, patient encounters with the general practice, and interventions. An episode of care (abbreviated to episode) is defined as ‘*a health problem in an individual from the first to the last encounter (contact) with a healthcare provider for that health problem*’.^12,14^ The time period of an episode of care was used to calculate the total duration of care. We excluded episodes where inclusion was based on medication prescription only (181 episodes in total), given that it was likely that these regarded preventive treatment of close contacts of a person diagnosed with scabies. All episodes have a diagnosis, which can be modified during the course of an episode as a result of diagnostic measures, therapeutic interventions, and referrals. Within a single episode, all interactions between the practice and patient are registered as encounters. This is any interaction between a patient and the GP or other members of the general practice. Encounter types were classified for this study in three categories and include the following: physical encounters (face-to-face consultation or home visit); administrative encounters (among others medical letters or repeat prescribing); and telephone encounters (telephone or email consultations). An episode may consist of one or more encounters.^14^ The GP decides whether a new encounter is part of an existing episode of care or part of a new episode of care.

The reason for encounter (RFE) represents a description of the demand of care. It is a spontaneous statement of the patient on why they visit the GP, and precedes the GP’s interpretation. The RFE may be presented as a symptom, a self-diagnosed disease, or a request for an intervention. RFEs are recorded regardless of the final diagnosis. If multiple RFEs are presented, these are all registered. We categorised the RFEs into four groups. The first group being ‘prevention, risk factor, or medication request’, with the corresponding ICPC codes, respectively, A98, A23, and *50 categorised together, in addition to the second group ‘scabies’ (S72) and the third group ‘itch’ (S02). All other RFEs were listed in the final group ‘other’. Patient delay is defined as the time interval, reported by the patient, during which they have experienced their symptoms. This time interval is registered at the first encounter of an episode.^14^ Doctor’s delay is defined as the time from the first encounter up to the scabies diagnosis by the GP. The time interval of patient and doctor’s delay was expressed in days and for doctor’s delay also in which encounter within an episode of care the scabies diagnosis was registered (for example, the first, second, or third, and so on encounter). An intervention is defined as an action performed by the GP as a result of the encounter. Interventions will be analysed for those limited to skin-related interventions to avoid misclassification of interventions for other health complaints not related to scabies within the same contact registration.

### Study analyses

Descriptive analyses were performed to address the incidences, characteristics, and trends in the use of care of patients with scabies between 2014–2021 and 2021–2023. Categorical variables were compared using the χ^2^ test or Fisher’s exact test. Statistical significance was set at *P*<0.05. Post hoc pairwise comparisons were conducted using Bonferroni correction to control for multiple comparisons. All statistical analyses were performed in R (version 4.1.3) and R studio (version 2022.02.1).^15,16^

## Results

### Increase in scabies-related episodes in the Netherlands

**Figure 1.**
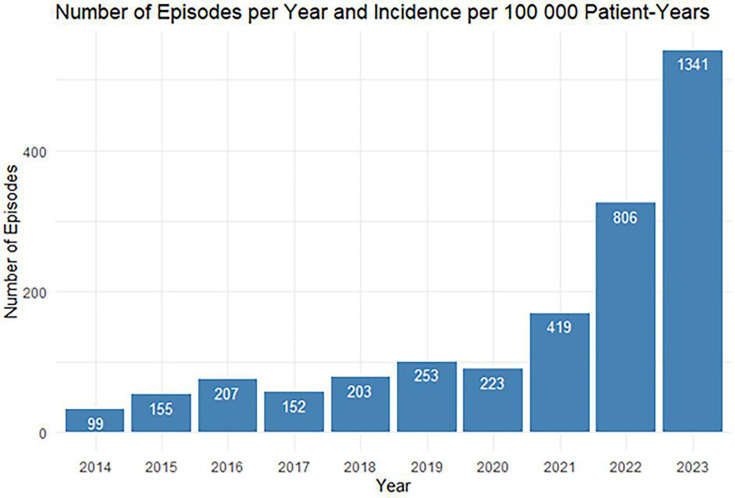
Scabies-related episodes per year between 2014 and 2023. The total number of episodes, based on the episode’s starting date, is shown per year in blue. The episode’s start date was defined as the patient’s first contact with the general practice for that episode. Incidence rate per 100 000 patient-years is shown per year in white

Between 2014 and 2023, a total of 1525 scabies-related episodes were recorded. The incidence of scabies-related episodes in primary care practices gradually rose from 99 to 223 episodes per 100 000 patient-years between 2014 and 2020 (Figure 1). From 2020 to 2023, an exponential increase was observed from 223 to 1341 scabies-related episodes per 100 000 patient-years. By 2023, the incidence of scabies-related episodes in primary care practices had risen more than 13-fold compared with 2014 (from 99 to 1341 per 100 000 patient-years). On average, 70 scabies-related episodes were documented annually between 2014 and 2020. This number increased to 345 episodes per year between 2021 and 2023.

This substantial increase prompted a comparative analysis of scabies incidence and the associated burden of care across two distinct time periods: 2014–2020 (low incidence) and 2021–2023 (high incidence). The proportion of patients who experienced more than one episode that started within the same year increased significantly between the two periods (4.0% between 2014–2020 versus 11.9% between 2021–2023, Fisher’s exact test *P*<0.001, Table 1).

**Table 1.** Patients with scabies-related episodes characteristics between 2014 and 2023

	**2014–2020 (** ***n*** **= 489)**			**2021–2023 (** ***n*** **= 1036)**			
	*n*	%	per 100 000 patient-years	*n*	%	per 100 000 patient-years	*P*-value
**Number of episodes per patient per year**	*n* = 467			*n* = 915			<0.001
1	448	95.9	172	806	88.1	666	^a^
2	16	3.4	6	97	10.6	80	^a^
3	3	0.6	1	12	1.3	10	ns
**Age,years**	*n* = 489			*n* = 1036			<0.001
0–3	13	2.7	5	22	2.1	18	ns
4–12	37	7.6	14	67	6.5	55	ns
13–16	28	5.7	11	36	3.5	30	^a^
17–25	186	38.0	71	562	54.2	464	^a^
26–45	112	22.9	43	199	19.2	164	ns
46–60	86	17.6	33	120	11.6	99	^a^
>60	27	5.5	10	30	2.9	25	^a^
**Sex**	*n* = 489			*n* = 1036			0.002
Female	221	45.2	85	558	53.9	461	^a^
Male	268	54.8	103	478	46.1	395	^a^

^a^Post-hoc analyses indicate that the difference between the two periods for a specific category is significant at *P*<0.05; whereas ns indicates a non-significant difference between the two periods (Bonferroni adjusted)

An increase in scabies-related episodes between the two time periods was observed in all age groups but was most notable in those aged 17–25 years (χ²[6, *n* = 1525] = 40.03, *P*<0.001, Table 1). The incidence in this group increased from 71 episodes per 100 000 patient-years between 2014–2020 to 464 episodes per 100 000 patient-years between 2021–2023. The female-to-male ratio increased from 0.82 (2014–2020) to 1.17 (2021–2023) episodes, respectively (χ²[1, *n* = 1525] = 9.99, *P* = 0.002, Table 1).

### Trends in patient and doctor delays

The nature of patient-reported complaints (RFE) shifted over time. During periods of low incidence, non-specific complaints such as ‘itch’ (ICPC S02) were frequently registered as the initial RFE in scabies-related episodes. In contrast, during high-incidence periods, more specific enquires such as ‘prevention’ (ICPC A98) (for example, ‘*Can I get preventive medication for scabies?*’), ‘risk factor’ (ICPC A23) (for example, *‘I have been in contact with someone with scabies’*), or ‘medication request’ (ICPC *50) (for example, *‘Can I get scabies medication?’*) and ‘scabies’ (S72) (for example, ‘*I think I have scabies*’) became predominant as the main reasons for seeking care (χ²[3, *n* = 1384] = 68.14, *P*<0.001, Table 2).

**Table 2.** Scabies-related episode characteristics between 2014 and 2023

	**2014–2020^a^**			**2021–2023**			
	***n***	**%**	**per 100 000** **patient-years**	***n***	**%**	**per 100 000** **patient-years**	***P*-value**
**Reason for encounter**							<0.001
Prevention, risk factor, or medication request (A98, A23, *50)	87	22.7	33	351	35.1	290	^d^
Scabies (S72)	84	21.9	32	332	33.2	274	^d^
Itch (S02)	110	28.7	42	143	14.3	118	^d^
Other	102	26.6	39	175	17.5	67	^d^
**Total**	383			1001			
**Patient’s delay, days^a^**							0.013
1–7	60	41.7	31	249	54.1	206	^d^
8–14	30	20.8	16	92	20.0	76	ns
15–21	11	7.6	6	34	7.4	28	ns
22–28	5	3.5	3	19	4.1	16	ns
>28	38	26.4	20	66	14.3	55	^d^
**Total**	144			460			
**Doctor’s delay, days^b^**							<0.001
0	322	83.9	123	848	92.6	700	^d^
1–7	19	4.9	7	21	2.3	17	^d^
8–14	6	1.6	2	7	0.8	6	ns
15–21	4	1.0	2	5	0.5	4	ns
22–28	5	1.3	2	2	0.2	2	^d^
>28	28	7.3	11	33	3.6	27	^d^
**Total**	384			916			
**Doctor’s delay^b^**							0.001
1st contact	326	84.9	125	846	92.3	699	^d^
2nd contact	25	6.5	10	32	3.5	26	^d^
3rd contact	10	2.6	4	18	2.0	15	ns
4th contact	7	1.8	3	8	0.9	7	ns
5th contact	8	2.1	3	4	0.4	3	^d^
>5 contacts	8	2.1	3	9	1.0	7	ns
**Total**	384			917			
**Total duration of care^c^**							<0.001
0–7 days (1 week)	225	58.6	86	465	50.6	384	^d^
8–30 days (1 month)	47	12.2	18	105	11.4	87	ns
31–60 days (2 months)	33	8.6	13	96	10.4	79	ns
61–90 days (3 months)	16	4.2	6	75	8.2	62	^d^
91–120 days (4 months)	5	1.3	2	43	4.7	36	^d^
121–150 days (5 months)	10	2.6	4	33	3.6	27	ns
151–180 days (6 months)	4	1.0	2	26	2.8	21	ns
>180 days (>6 months)	44	11.5	17	76	8.3	63	ns
**Total**	384			919			

^a^Patient delay data have been registered from 2016 onwards. ^b^Only patients with scabies diagnosis code S72 were included. ^c^Episodes included based on medication prescription only were excluded from analysis, ^d^Post-hoc analyses indicate that the difference between the two periods for a specific category is significant at *P*<0.05; whereas ns indicates a non-significant difference between the two periods (Bonferroni adjusted)

Patient delay, defined as the time between the onset of scabies-related symptoms and the first contact with a general practice, has been systematically recorded in FaMe-Net since 2016 and showed a significant change between the two periods (χ²[4, *n* = 604] = 12.73, *P* = 0.013). Notably, the proportion of patients reporting delays exceeding 28 days decreased significantly from 26.4% during the 2016–2020 period to 14.3% in the 2021–2023 period. At the same time, more patients sought care within 7 days, with the proportion increasing from 41.7% in 2016–2020 to 54.1% in 2020–2023 (Table 2).

Doctor delay, defined as the time from a patient’s first contact with the general practice to a registered scabies diagnosis, also showed marked improvement over time. In 2014–2020, 84.9% of diagnoses were made during the initial consultation compared with 92.3% between 2021 and 2023 (Fisher’s exact test *P* = 0.001) (Table 2).

Finally, we also analysed the total duration of care. The total duration of care remained comparable between the two periods. The majority of episodes lasted ≤1 week during the low and high-incidence period (58.6% versus 50.6%, respectively, Fisher’s exact test *P*<0.001, Table 2).

### Rising burden of care in general practices

To assess the burden of care associated with scabies-related episodes in general practices over time, the total number of encounters during an episode was calculated for the entire study period. Between 2014 and 2023, a total of 4555 encounters were recorded. The annual number of contacts increased parallel with the increasing number of scabies-related episodes, rising from 614 contacts per 100 000 patient-years during 2014–2020 to 2438 contacts during 2021–2023. Despite this increase, the average number of contact moments per episode remained consistent across these periods (χ²[3, *n* = 1476] = 5.33, *P* =0.149).

Over time, an increase in all type of encounters with general practices was observed. However, a noticeable shift occurred in the proportion of telephone contacts from the low-incidence period (27.0%) to the high-incidence period (43.7%), (χ²[2, *n* = 4554] = 124,65, *P*<0.001, Table 3).

**Table 3.** Contact characteristics between 2014 and 2023

	**2014–2020**			**2021–2023**			
	*n*	%	per 100 000 patient-years	*n*	%	per 100 000 patient-years	*P*-value
**Number of encounters**	1604		614	2951		2438	
**Type of encounter**							<0.001
Physical	653	40.7	250	896	30.4	740	^a^
Administrative	518	32.3	198	764	25.9	631	^a^
Telephone	433	27.0	166	1290	43.7	1066	^a^
**Total**	1604			2950			
**Type of interventions**							<0.001
No intervention	557	36.9	213	518	14.4	428	^a^
Physical examination	374	24.8	143	660	18.4	545	^a^
Medication prescription or request	792	52.5	303	2026	56.4	1674	^a^
Health education or advice	245	16.2	94	769	21.4	635	^a^
Referral to specialist or hospital	46	3.0	18	85	2.4	70	ns
Other	52	3.4	20	53	1.5	44	^a^
**Total**	1509			3593			

^a^Post-hoc analyses indicate that the difference between the two periods for a specific category is significant at *P*<0.05; whereas ns indicates a non-significant difference between the two periods (Bonferroni adjusted)

Additionally, we analysed the number and type of interventions as a result of the encounter. The overall percentage of encounters with an intervention increased significantly over time: where 36.9% of contacts between 2014 and 2020 had no registered intervention, this decreased to 14.4% of contacts in 2021–2023 (Bonferroni corrected *P*<0.001, Table 3). With respect to the intervention type, the proportion of medical prescriptions increased significantly during the high-incidence period, rising from 52.5% to 56.4% (Bonferroni corrected *P*<0.001, Table 3). Furthermore, we calculated the number of referrals per encounter (Table 3). We also calculated the number of referrals per patient to dermatologists. This increased from 12 per 100 000 patient-years in 2014–2020 to 66 per 100 000 patient-years in 2021–2023.

## Discussion

### Summary

This study examines the epidemiology and burden of care of scabies-related episodes in selected Dutch general practices between 2014 and 2023. To our knowledge, this is the first study to quantify the burden of scabies-related care in general practices. Our findings highlight an unprecedented increase in scabies-related episodes and an increased burden of care, including more consultations and interventions, in general practices.

### Strengths and limitations

The main strength of this study was the use of a highly reliable primary care database, FaMe-Net. The systematic recording of health problems, interventions, and care processes within FaMe-Net provides detailed insights into episodes of care in primary care settings. Although registration errors of healthcare providers cannot be excluded, FaMe-Net performs systematic quality checks of data, provides feedback to GPs, and offers continuous training and quality control programmes for healthcare providers to ensure a high-quality database.^17^

The main limitation of this study was that data were derived from a limited number of general practices and from a single region within the Netherlands, which may affect the generalisability of the findings. However, the observed increased incidence aligns with national trends,^9,18^ suggesting that these findings reflect broader patterns. We therefore do not anticipate substantial differences in the associated burden of care at the national level.

### Comparison with existing literature

The rising incidence of scabies-related episodes observed in this study is consistent with trends reported in other high-income countries.^10^ These findings are supported by various data sources, all of which document similar increases in scabies cases. However, accurately quantifying the true incidence remains challenging, as scabies is not routinely a notifiable disease in most countries, including the Netherlands. The topical scabicide (permethrin) is also available over the counter in the Netherlands.^19^ Consequently, the actual burden of scabies in the Dutch population is likely underreported and remains uncertain. Although we observed that all age groups were affected, our findings align with previous reports showing the highest proportion of consultations among student age groups.^7^ Moreover, scabies episodes were more frequently reported by females than males, although the literature on the male-to-female ratio remains inconsistent.^20^

### Implications for research and practice

We have no clear explanation for the ongoing exponential increase in scabies-related episodes observed from 2021 onwards. Likely drivers of ongoing transmission include treatment challenges, such as medication resistance, reinfection owing to incomplete decontamination of the patient’s environment, failure to treat close contacts adequately, and treatment errors.^6,21–24^ Their respective roles in ongoing transmission require further research with the purpose of informing intervention strategies. The impact of COVID-19 on scabies is unclear, but scabies cases rose even as other infectious diseases declined. One hypothesis is that changes in social behaviours, such as household sharing spaces for longer periods of time, increased sexual activity during lockdown, and working from home, may have temporarily contributed to transmission, but these do not explain the ongoing increasing trend.^10,25,26^

Our findings indicate that scabies is being diagnosed more quickly than in previous years. This improvement may be attributed to increased clinical experience and heightened awareness among GPs and the general public, which could facilitate earlier recognition. The diagnoses of scabies is predominantly based on clinical evaluation, which has inherently low specificity owing to the often non-specific nature of its clinical presentation. This dependence on clinical judgement makes the accuracy of the diagnosis heavily reliant on the experience and awareness of the GP. Hence, without the availability of better diagnostic tools, this heightened awareness might also lead to increased scabies-related consultations and false-positive diagnoses, increasing the burden of care even further.

Taken together, these findings emphasise the complex nature of scabies epidemiology and highlight the need for improved surveillance, more effective diagnostic strategies, and targeted public health interventions to halt transmission and limit the pressure on a burdened primary healthcare system.^27^ Interventions may prompt timely care through awareness, especially in high-incidence groups. Scabies is treatable; early, improved diagnosis helps prevent spread. In addition, adherence to treatment and treating close contacts are key to controlling scabies, especially in crowded settings such as student housing or care centres. In the Netherlands, a limited number of PHS have successfully established specialised scabies consultation hours — either in person or via telephone — for individuals with treatment challenges.

In conclusion, the rising burden of scabies-related consultations in general practice highlights it as an urgent public health concern. We recommend strengthening collaboration between public health specialists and GPs to address the surge in scabies cases effectively. With no preventive measures beyond treating close contacts, increasing awareness for early recognition is crucial. Additionally, developing rapid, cost-effective diagnostic tools is essential for timely diagnosis and treatment. Managing scabies requires a multifaceted strategy that integrates clinical, public health, and policy-level solutions to mitigate the impact on a resource-limited healthcare system and affected populations.

## Data

The dataset relied on in this article is available from the corresponding author on reasonable request.
